# 5-Acetyl-4-(2-chloro­phen­yl)-6-methyl-3,4-dihydro­pyrimidin-2(1*H*)-one

**DOI:** 10.1107/S1600536808039366

**Published:** 2008-11-29

**Authors:** N. Anuradha, A. Thiruvalluvar, K. Pandiarajan, S. Chitra, R. J. Butcher

**Affiliations:** a PG Research Department of Physics, Rajah Serfoji Government College (Autonomous), Thanjavur 613 005, Tamil Nadu, India; b Department of Chemistry, Annamalai University, Annamalai Nagar 608 002, Tamilnadu, India; c Department of Chemistry, Howard University, 525 College Street NW, Washington, DC 20059, USA

## Abstract

In the title mol­ecule, C_13_H_13_ClN_2_O_2_, the heterocyclic ring adopts a flattened boat conformation with the plane through the four coplanar atoms making a dihedral angle of 89.16 (5)° with the benzene ring, which adopts an axial orientation. The carbonyl, acetyl and methyl groups each have an equatorial orientation. In the crystal structure, inter­molecular N—H⋯O hydrogen bonds lead to a tape motif. The H atoms of the methyl group at position 6 are disordered over two positions of opposite orientation.

## Related literature

For the biological applications of dihydro­pyrimidinone derivatives, see: Ghorab *et al.* (2000 ▶); Kappe (1993 ▶, 2000 ▶); Kappe *et al.* (1997 ▶); Rovnyak *et al.* (1992 ▶, 1995 ▶); Shivarama Holla *et al.* (2004 ▶).
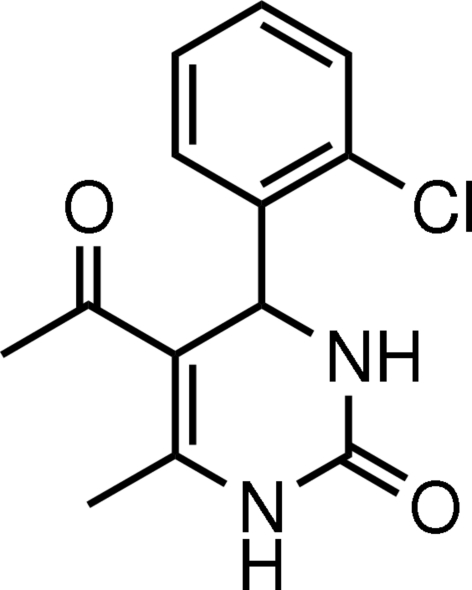


## Experimental

### 

#### Crystal data

C_13_H_13_ClN_2_O_2_
*M* *_r_* = 264.70
Orthorhombic, 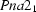

*a* = 14.5364 (8) Å
*b* = 12.1587 (5) Å
*c* = 7.0780 (4) Å
*V* = 1250.99 (11) Å^3^
*Z* = 4
Mo *K*α radiation
μ = 0.30 mm^−1^
*T* = 296 (2) K
0.58 × 0.22 × 0.16 mm

#### Data collection

Bruker APEXII CCD diffractometer
Absorption correction: multi-scan (*SADABS*; Bruker, 2004 ▶) *T*_min_ = 0.845, *T*_max_ = 0.954
22043 measured reflections
3637 independent reflections
3087 reflections with *I* > 2σ(*I*)
*R*_int_ = 0.043

#### Refinement

*R*[*F* ^2^ > 2σ(*F* ^2^)] = 0.037
*wR*(*F* ^2^) = 0.090
*S* = 1.03
3637 reflections
172 parameters
1 restraint
H atoms treated by a mixture of independent and constrained refinement
Δρ_max_ = 0.20 e Å^−3^
Δρ_min_ = −0.22 e Å^−3^
Absolute structure: Flack (1983 ▶), 1654 Friedel pairs
Flack parameter: 0.01 (6)

### 

Data collection: *APEX2* (Bruker, 2004 ▶); cell refinement: *APEX2*; data reduction: *SAINT-NT* (Bruker, 2004 ▶); program(s) used to solve structure: *SHELXS97* (Sheldrick, 2008 ▶); program(s) used to refine structure: *SHELXL97* (Sheldrick, 2008 ▶); molecular graphics: *ORTEP-3* (Farrugia, 1997 ▶); software used to prepare material for publication: *PLATON* (Spek, 2003 ▶).

## Supplementary Material

Crystal structure: contains datablocks global, I. DOI: 10.1107/S1600536808039366/tk2333sup1.cif

Structure factors: contains datablocks I. DOI: 10.1107/S1600536808039366/tk2333Isup2.hkl

Additional supplementary materials:  crystallographic information; 3D view; checkCIF report

## Figures and Tables

**Table 1:** Hydrogen-bond geometry (Å, °)

*D*—H⋯*A*	*D*—H	H⋯*A*	*D*⋯*A*	*D*—H⋯*A*
N1—H1⋯O2^i^	0.80 (2)	2.05 (2)	2.8386 (18)	170.6 (19)
N3—H3⋯Cl1	0.83 (2)	2.748 (18)	3.2005 (15)	116.2 (14)
N3—H3⋯O2^ii^	0.83 (2)	2.18 (2)	2.9627 (18)	158.6 (16)
C4—H4⋯O15	0.98	2.35	2.712 (2)	101
C16—H16*A*⋯O2^iii^	0.96	2.51	3.421 (2)	159
C45—H45⋯O15^iv^	0.93	2.55	3.257 (3)	133
C16—H16*C*⋯*Cg*^iii^	0.96	2.86	3.699 (2)	147

Symmetry codes: (i) 
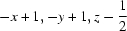
; (ii) 
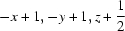
; (iii) 
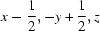
; (iv) 
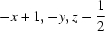
. Cg is the centroid of the C41–C46 ring.

**Fig. 1.**
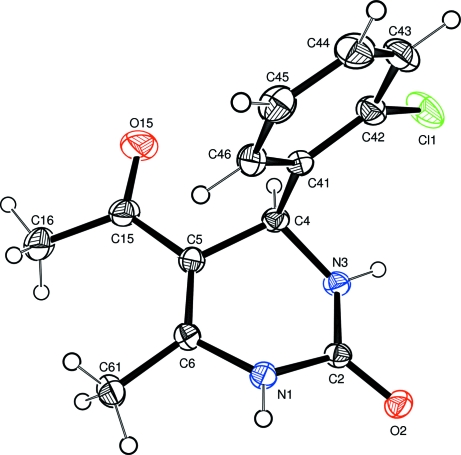
The molecular structure of (I), showing the atom-numbering scheme and displacement ellipsoids drawn at the 30% probability level. H atoms are shown as small spheres of arbitrary radius.

**Fig. 2.**
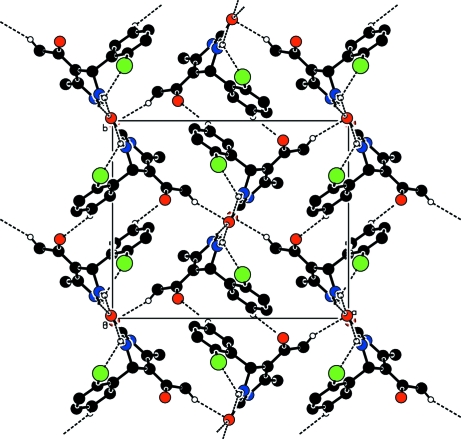
The crystal packing of (I), viewed down the c axis. Dashed lines indicate intermolecular contacts. H atoms not involved in hydrogen bonding have been omitted.

## supplementary crystallographic information

### Comment

Dihydropyrimidinone derivatives exhibit a wide range of biological effects including anti-fungal, anti-viral, anti-cancer, anti-bacterial, and anti-inflammatory activities (Kappe, 2000; Ghorab *et al.*, 2000; Shivarama Holla *et al.*, 2004). Some dihydropyrimidinones exhibit anti-tumour properties (Kappe, 1993). In addition, these compounds have emerged as the integral backbones of several calcium channel blockers (Rovnyak *et al.*, 1995), antagonists (Kappe *et al.*, 1997) and anti-hypertensive agents (Rovnyak *et al.*, 1992).

In the title molecule, C_13_H_13_ClN_2_O_2_, (I) & Fig. 1, the heterocyclic ring adopts a flattened boat conformation with the plane through the four co- planar atoms (N3, C2, C5 and C6) forming a dihedral angle of 89.16 (5)° with the benzene ring, which is in an axial orientation. The carbonyl, acetyl and methyl groups are each in an equatorial orientation. Intermolecular N1—H1···O2, N3—H3···O2, C16—H16A···O2 and C45—H45···O15 interactions, and N3—H3···Cl1 and C4—H4···O15 intramolecular contacts are found, Table 1. The N—H···O hydrogen bonding leads to the formation of tapes. Further, a C16—H16C···π interaction is also found involving the benzene (C41—C46) ring. Fig. 2 shows a view of the unit-cell contents.

### Experimental

A solution of acetylacetone (1.0012 g, 0.01 mol), 2-chlorobenzaldehyde (1.4057 g, 0.01 mol) and urea (0.90 g, 0.015 mol) was heated under reflux in the presence of calcium chloride (0.1109 g, 0.001 mol) for 5 h (monitored by TLC). After completion of the reaction, the reaction mixture was cooled to room temperature and poured into crushed ice. The solid product was filtered under suction and purified by column chromatography on silica gel. Elution with 1:1 (benzene:ethyl acetate *v*/*v*) gave the product in the pure form. Yield 0.79 g (88%).

### Refinement

The N-bound H atoms were located in a difference Fourier map and refined isotropically, see Table 1 for bond distances. The remaining H atoms were positioned geometrically and allowed to ride on their parent atoms, with C—H = 0.93 - 0.98 Å and *U*_iso_(H) = 1.2 - 1.5 times *U*_eq_(C). The H atoms bound to the C6-methyl group were found to be disordered over two positions with equal weight.

### Crystal data

**Table:**

C_13_H_13_ClN_2_O_2_	*D*_x_ = 1.406 Mg m^−^^3^
*M**_r_* = 264.70	Melting point: 555.5 K
Orthorhombic, *P**n**a*2_1_	Mo *K*α radiation λ = 0.71073 Å
Hall symbol: P 2c -2n	Cell parameters from 6857 reflections
*a* = 14.5364 (8) Å	θ = 2.8–25.4º
*b* = 12.1587 (5) Å	µ = 0.30 mm^−^^1^
*c* = 7.0780 (4) Å	*T* = 296 (2) K
*V* = 1250.99 (11) Å^3^	Needle, colourless
*Z* = 4	0.58 × 0.22 × 0.16 mm
*F*_000_ = 552	

### Data collection

**Table:**

Bruker APEXII CCD diffractometer	3637 independent reflections
Radiation source: fine-focus sealed tube	3087 reflections with *I* > 2σ(*I*)
Monochromator: graphite	*R*_int_ = 0.043
*T* = 296(2) K	θ_max_ = 30.1º
φ and ω scans	θ_min_ = 2.2º
Absorption correction: multi-scan(SADABS; Bruker, 2004)	*h* = −20→20
*T*_min_ = 0.845, *T*_max_ = 0.954	*k* = −17→16
22043 measured reflections	*l* = −9→9

### Refinement

**Table:**

Refinement on *F*^2^	Hydrogen site location: inferred from neighbouring sites
Least-squares matrix: full	H atoms treated by a mixture of independent and constrained refinement
*R*[*F*^2^ > 2σ(*F*^2^)] = 0.037	*w* = 1/[σ^2^(*F*_o_^2^) + (0.0429*P*)^2^ + 0.1683*P*] where *P* = (*F*_o_^2^ + 2*F*_c_^2^)/3
*wR*(*F*^2^) = 0.090	(Δ/σ)_max_ = 0.001
*S* = 1.03	Δρ_max_ = 0.20 e Å^−^^3^
3637 reflections	Δρ_min_ = −0.22 e Å^−^^3^
172 parameters	Extinction correction: none
1 restraint	Absolute structure: Flack (1983), 1654 Friedel pairs
Primary atom site location: structure-invariant direct methods	Flack parameter: 0.01 (6)
Secondary atom site location: difference Fourier map	

### Special details

**Table:**

Geometry. Bond distances, angles *etc*. have been calculated using the rounded fractional coordinates. All su's are estimated from the variances of the (full) variance-covariance matrix. The cell e.s.d.'s are taken into account in the estimation of distances, angles and torsion angles
Refinement. Refinement of *F*^2^ against ALL reflections. The weighted *R*-factor *wR* and goodness of fit *S* are based on *F*^2^, conventional *R*-factors *R* are based on *F*, with *F* set to zero for negative *F*^2^. The threshold expression of *F*^2^ > 2σ(*F*^2^) is used only for calculating *R*-factors(gt) *etc*. and is not relevant to the choice of reflections for refinement. *R*-factors based on *F*^2^ are statistically about twice as large as those based on *F*, and *R*- factors based on ALL data will be even larger.

### Fractional atomic coordinates and isotropic or equivalent isotropic displacement parameters (Å^2^)

**Table:**

	*x*	*y*	*z*	*U*_iso_*/*U*_eq_	Occ. (<1)
Cl1	0.55079 (4)	0.22136 (5)	1.08117 (7)	0.0597 (2)	
O2	0.50642 (8)	0.51312 (9)	0.62182 (16)	0.0343 (3)	
O15	0.27723 (11)	0.09996 (13)	0.7672 (3)	0.0774 (6)	
N1	0.42438 (10)	0.38746 (11)	0.4557 (2)	0.0317 (4)	
N3	0.44410 (10)	0.36578 (10)	0.7731 (2)	0.0307 (4)	
C2	0.46183 (10)	0.42625 (12)	0.6203 (2)	0.0272 (4)	
C4	0.41461 (11)	0.25076 (12)	0.7555 (2)	0.0287 (4)	
C5	0.34298 (9)	0.24351 (12)	0.6009 (2)	0.0292 (4)	
C6	0.35395 (10)	0.31050 (12)	0.4495 (2)	0.0292 (4)	
C15	0.27134 (12)	0.16018 (14)	0.6294 (3)	0.0411 (5)	
C16	0.19130 (12)	0.14572 (17)	0.4994 (4)	0.0527 (7)	
C41	0.49732 (11)	0.17499 (12)	0.7223 (2)	0.0289 (4)	
C42	0.56389 (12)	0.15910 (13)	0.8612 (3)	0.0375 (5)	
C43	0.64096 (14)	0.09425 (16)	0.8322 (3)	0.0486 (6)	
C44	0.65258 (14)	0.04277 (16)	0.6600 (3)	0.0484 (6)	
C45	0.58767 (13)	0.05448 (15)	0.5208 (3)	0.0430 (5)	
C46	0.51081 (12)	0.11989 (13)	0.5529 (2)	0.0351 (5)	
C61	0.30143 (13)	0.31541 (15)	0.2672 (3)	0.0423 (5)	
H1	0.4383 (13)	0.4194 (16)	0.362 (3)	0.039 (5)*	
H3	0.4662 (12)	0.3844 (14)	0.876 (3)	0.028 (5)*	
H4	0.38548	0.22909	0.87471	0.0345*	
H16A	0.15191	0.08874	0.54708	0.0791*	
H16B	0.21307	0.12573	0.37608	0.0791*	
H16C	0.15749	0.21339	0.49164	0.0791*	
H43	0.68435	0.08551	0.92754	0.0584*	
H44	0.70457	0.00002	0.63836	0.0581*	
H45	0.59505	0.01882	0.40558	0.0516*	
H46	0.46706	0.12694	0.45776	0.0421*	
H61A	0.32656	0.37231	0.18861	0.0635*	0.500
H61B	0.23793	0.33107	0.29304	0.0635*	0.500
H61C	0.30619	0.24599	0.20333	0.0635*	0.500
H61D	0.25389	0.26060	0.26804	0.0635*	0.500
H61E	0.34252	0.30185	0.16361	0.0635*	0.500
H61F	0.27426	0.38692	0.25332	0.0635*	0.500

### Atomic displacement parameters (Å^2^)

**Table:**

	*U*^11^	*U*^22^	*U*^33^	*U*^12^	*U*^13^	*U*^23^
Cl1	0.0743 (3)	0.0725 (3)	0.0323 (2)	0.0214 (3)	−0.0192 (2)	−0.0082 (2)
O2	0.0433 (6)	0.0301 (5)	0.0294 (6)	−0.0071 (4)	−0.0026 (5)	0.0000 (4)
O15	0.0606 (9)	0.0728 (10)	0.0989 (14)	−0.0278 (8)	−0.0217 (9)	0.0522 (10)
N1	0.0394 (7)	0.0341 (6)	0.0216 (6)	−0.0083 (5)	−0.0014 (6)	0.0039 (5)
N3	0.0430 (8)	0.0278 (6)	0.0212 (6)	−0.0006 (5)	−0.0028 (6)	−0.0001 (5)
C2	0.0303 (7)	0.0269 (6)	0.0244 (7)	0.0021 (5)	0.0006 (5)	0.0002 (5)
C4	0.0333 (7)	0.0282 (6)	0.0247 (7)	−0.0022 (6)	0.0018 (6)	0.0040 (5)
C5	0.0250 (6)	0.0306 (7)	0.0321 (8)	−0.0014 (5)	0.0002 (6)	0.0029 (6)
C6	0.0276 (7)	0.0312 (7)	0.0289 (8)	0.0005 (6)	−0.0009 (6)	−0.0001 (6)
C15	0.0309 (8)	0.0366 (8)	0.0558 (12)	−0.0015 (6)	−0.0004 (8)	0.0099 (8)
C16	0.0332 (9)	0.0493 (10)	0.0757 (15)	−0.0123 (8)	−0.0068 (9)	0.0098 (10)
C41	0.0307 (8)	0.0268 (6)	0.0292 (8)	−0.0029 (5)	−0.0010 (6)	0.0044 (5)
C42	0.0436 (9)	0.0360 (8)	0.0328 (8)	0.0025 (7)	−0.0059 (7)	0.0011 (7)
C43	0.0448 (10)	0.0489 (10)	0.0522 (11)	0.0116 (8)	−0.0135 (9)	0.0044 (9)
C44	0.0428 (10)	0.0409 (9)	0.0616 (13)	0.0128 (8)	0.0011 (9)	−0.0012 (9)
C45	0.0459 (10)	0.0367 (8)	0.0465 (10)	0.0027 (7)	0.0052 (8)	−0.0087 (7)
C46	0.0381 (8)	0.0338 (7)	0.0334 (9)	−0.0009 (6)	−0.0031 (7)	−0.0031 (6)
C61	0.0423 (9)	0.0493 (10)	0.0354 (8)	−0.0078 (8)	−0.0091 (8)	0.0036 (8)

### Geometric parameters (Å, °)

**Table:**

Cl1—C42	1.742 (2)	C43—C44	1.381 (3)
O2—C2	1.2393 (18)	C44—C45	1.372 (3)
O15—C15	1.223 (3)	C45—C46	1.390 (3)
N1—C2	1.370 (2)	C4—H4	0.9800
N1—C6	1.388 (2)	C16—H16A	0.9600
N3—C2	1.333 (2)	C16—H16B	0.9600
N3—C4	1.4680 (19)	C16—H16C	0.9600
N1—H1	0.80 (2)	C43—H43	0.9300
N3—H3	0.83 (2)	C44—H44	0.9300
C4—C41	1.533 (2)	C45—H45	0.9300
C4—C5	1.513 (2)	C46—H46	0.9300
C5—C6	1.355 (2)	C61—H61A	0.9600
C5—C15	1.467 (2)	C61—H61B	0.9600
C6—C61	1.501 (3)	C61—H61C	0.9600
C15—C16	1.494 (3)	C61—H61D	0.9600
C41—C46	1.387 (2)	C61—H61E	0.9600
C41—C42	1.393 (2)	C61—H61F	0.9600
C42—C43	1.385 (3)		
			
Cl1···N3	3.2005 (15)	C46···H16C^v^	2.9700
Cl1···C46^i^	3.6067 (15)	C61···H16B	2.7500
Cl1···O2^ii^	3.3462 (13)	C61···H16C	2.9100
Cl1···H4	2.8100	H1···H61A	2.1100
Cl1···H46^i^	3.1500	H1···H61E	2.4400
Cl1···H3	2.748 (18)	H1···H61F	2.5400
O2···Cl1^iii^	3.3462 (13)	H1···O2^iii^	2.05 (2)
O2···N1^ii^	2.8386 (18)	H1···C2^iii^	2.93 (2)
O2···N3^iii^	2.9627 (18)	H3···Cl1	2.748 (18)
O15···C45^iv^	3.257 (3)	H3···C42	3.087 (17)
O15···C41	3.342 (2)	H3···O2^ii^	2.18 (2)
O2···H61A^ii^	2.8400	H3···C2^ii^	3.064 (19)
O2···H1^ii^	2.05 (2)	H4···Cl1	2.8100
O2···H3^iii^	2.18 (2)	H4···O15	2.3500
O2···H16A^v^	2.5100	H4···H61E^i^	2.3100
O15···H4	2.3500	H16A···O2^vii^	2.5100
O15···H44^iv^	2.9100	H16A···C2^vii^	2.8200
O15···H45^iv^	2.5500	H16B···C6	3.0800
O15···H61F^vi^	2.7000	H16B···C61	2.7500
N1···C41	3.370 (2)	H16B···H61B	2.5900
N1···O2^iii^	2.8386 (18)	H16B···H61C	2.3400
N3···Cl1	3.2005 (15)	H16B···H61D	1.9000
N3···O2^ii^	2.9627 (18)	H16B···H44^viii^	2.5700
C2···C16^v^	3.553 (2)	H16C···C61	2.9100
C6···C46	3.333 (2)	H16C···H61B	2.3200
C15···C46	3.557 (2)	H16C···H61D	2.1900
C16···C2^vii^	3.553 (2)	H16C···C45^vii^	3.0100
C16···C61	3.086 (3)	H16C···C46^vii^	2.9700
C41···O15	3.342 (2)	H44···O15^viii^	2.9100
C41···N1	3.370 (2)	H44···H16B^iv^	2.5700
C42···C45^iv^	3.588 (3)	H45···O15^viii^	2.5500
C45···C42^viii^	3.588 (3)	H45···C41^viii^	3.0100
C45···O15^viii^	3.257 (3)	H46···Cl1^ix^	3.1500
C46···Cl1^ix^	3.6067 (15)	H46···C5	2.5100
C46···C15	3.557 (2)	H46···C6	2.7700
C46···C6	3.333 (2)	H61A···H1	2.1100
C61···C16	3.086 (3)	H61A···O2^iii^	2.8400
C2···H16A^v^	2.8200	H61B···C16	2.7700
C2···H1^ii^	2.93 (2)	H61B···H16B	2.5900
C2···H3^iii^	3.064 (19)	H61B···H16C	2.3200
C5···H46	2.5100	H61B···C45^vii^	3.0500
C6···H46	2.7700	H61C···C16	2.9400
C6···H16B	3.0800	H61C···H16B	2.3400
C15···H61D	2.8500	H61D···C15	2.8500
C16···H61C	2.9400	H61D···C16	2.3400
C16···H61D	2.3400	H61D···H16B	1.9000
C16···H61B	2.7700	H61D···H16C	2.1900
C41···H45^iv^	3.0100	H61E···H1	2.4400
C42···H3	3.087 (17)	H61E···H4^ix^	2.3100
C45···H61B^v^	3.0500	H61F···H1	2.5400
C45···H16C^v^	3.0100	H61F···O15^x^	2.7000
			
C2—N1—C6	123.53 (13)	C15—C16—H16B	109.00
C2—N3—C4	120.87 (13)	C15—C16—H16C	109.00
C2—N1—H1	116.1 (14)	H16A—C16—H16B	109.00
C6—N1—H1	119.4 (14)	H16A—C16—H16C	109.00
C2—N3—H3	119.2 (12)	H16B—C16—H16C	110.00
C4—N3—H3	116.7 (12)	C42—C43—H43	120.00
O2—C2—N3	124.35 (14)	C44—C43—H43	120.00
N1—C2—N3	115.05 (13)	C43—C44—H44	120.00
O2—C2—N1	120.58 (13)	C45—C44—H44	120.00
N3—C4—C5	108.53 (12)	C44—C45—H45	120.00
N3—C4—C41	110.89 (13)	C46—C45—H45	120.00
C5—C4—C41	113.20 (12)	C41—C46—H46	119.00
C4—C5—C15	115.43 (13)	C45—C46—H46	119.00
C6—C5—C15	127.40 (14)	C6—C61—H61A	109.00
C4—C5—C6	117.12 (13)	C6—C61—H61B	109.00
N1—C6—C5	117.84 (13)	C6—C61—H61C	109.00
N1—C6—C61	112.07 (13)	C6—C61—H61D	109.00
C5—C6—C61	130.09 (14)	C6—C61—H61E	109.00
C5—C15—C16	123.34 (17)	C6—C61—H61F	109.00
O15—C15—C5	118.27 (17)	H61A—C61—H61B	109.00
O15—C15—C16	118.39 (17)	H61A—C61—H61C	109.00
C4—C41—C46	122.25 (14)	H61A—C61—H61D	141.00
C42—C41—C46	116.41 (15)	H61A—C61—H61E	56.00
C4—C41—C42	121.34 (13)	H61A—C61—H61F	56.00
Cl1—C42—C41	119.65 (13)	H61B—C61—H61C	109.00
Cl1—C42—C43	117.92 (15)	H61B—C61—H61D	56.00
C41—C42—C43	122.43 (18)	H61B—C61—H61E	141.00
C42—C43—C44	119.20 (19)	H61B—C61—H61F	56.00
C43—C44—C45	120.20 (19)	H61C—C61—H61D	56.00
C44—C45—C46	119.66 (18)	H61C—C61—H61E	56.00
C41—C46—C45	122.08 (16)	H61C—C61—H61F	141.00
N3—C4—H4	108.00	H61D—C61—H61E	109.00
C5—C4—H4	108.00	H61D—C61—H61F	109.00
C41—C4—H4	108.00	H61E—C61—H61F	109.00
C15—C16—H16A	109.00		
			
C6—N1—C2—O2	−161.31 (14)	C15—C5—C6—N1	177.02 (15)
C6—N1—C2—N3	16.9 (2)	C15—C5—C6—C61	−3.7 (3)
C2—N1—C6—C5	−23.4 (2)	C4—C5—C15—O15	−3.9 (2)
C2—N1—C6—C61	157.25 (15)	C4—C5—C15—C16	175.71 (16)
C4—N3—C2—O2	−162.51 (15)	C6—C5—C15—O15	173.16 (17)
C4—N3—C2—N1	19.4 (2)	C6—C5—C15—C16	−7.3 (3)
C2—N3—C4—C5	−44.08 (19)	C4—C41—C42—Cl1	2.9 (2)
C2—N3—C4—C41	80.86 (17)	C4—C41—C42—C43	−177.59 (16)
N3—C4—C5—C6	35.79 (18)	C46—C41—C42—Cl1	−177.91 (12)
N3—C4—C5—C15	−146.88 (14)	C46—C41—C42—C43	1.6 (2)
C41—C4—C5—C6	−87.77 (16)	C4—C41—C46—C45	177.56 (15)
C41—C4—C5—C15	89.56 (16)	C42—C41—C46—C45	−1.6 (2)
N3—C4—C41—C42	66.67 (18)	Cl1—C42—C43—C44	179.21 (15)
N3—C4—C41—C46	−112.46 (16)	C41—C42—C43—C44	−0.3 (3)
C5—C4—C41—C42	−171.08 (14)	C42—C43—C44—C45	−1.1 (3)
C5—C4—C41—C46	9.8 (2)	C43—C44—C45—C46	1.0 (3)
C4—C5—C6—N1	−6.0 (2)	C44—C45—C46—C41	0.4 (3)
C4—C5—C6—C61	173.24 (15)		

Symmetry codes: (i) *x*, *y*, *z*+1; (ii) −*x*+1, −*y*+1, *z*+1/2; (iii) −*x*+1, −*y*+1, *z*−1/2; (iv) −*x*+1, −*y*, *z*+1/2; (v) *x*+1/2, −*y*+1/2, *z*; (vi) −*x*+1/2, *y*−1/2, *z*+1/2; (vii) *x*−1/2, −*y*+1/2, *z*; (viii) −*x*+1, −*y*, *z*−1/2; (ix) *x*, *y*, *z*−1; (x) −*x*+1/2, *y*+1/2, *z*−1/2.

### Hydrogen-bond geometry (Å, °)

**Table:**

*D*—H···*A*	*D*—H	H···*A*	*D*···*A*	*D*—H···*A*
N1—H1···O2^iii^	0.80 (2)	2.05 (2)	2.8386 (18)	170.6 (19)
N3—H3···Cl1	0.83 (2)	2.748 (18)	3.2005 (15)	116.2 (14)
N3—H3···O2^ii^	0.83 (2)	2.18 (2)	2.9627 (18)	158.6 (16)
C4—H4···O15	0.98	2.35	2.712 (2)	101
C16—H16A···O2^vii^	0.96	2.51	3.421 (2)	159
C45—H45···O15^viii^	0.93	2.55	3.257 (3)	133
C16—H16C···Cg^vii^	0.96	2.86	3.699 (2)	147

Symmetry codes: (iii) −*x*+1, −*y*+1, *z*−1/2; (ii) −*x*+1, −*y*+1, *z*+1/2; (vii) *x*−1/2, −*y*+1/2, *z*; (viii) −*x*+1, −*y*, *z*−1/2.

## References

[bb1] Bruker (2004). *APEX2*, *SAINT-NT* and *SADABS* Bruker AXS Inc., Madison, Wisconsin, USA.

[bb2] Farrugia, L. J. (1997). *J. Appl. Cryst.* **30**, 565.

[bb3] Flack, H. D. (1983). *Acta Cryst.* A**39**, 876–881.

[bb4] Ghorab, M. M., Abdel-Gawad, S. M. & El-Gaby, M. S. A. (2000). *Farmaco*, **55**, 249–255. 10.1016/s0014-827x(00)00029-x 10966155

[bb5] Kappe, C. O. (1993). *Tetrahedron*, **49**, 6937–6963.

[bb6] Kappe, C. O. (2000). *Eur. J. Med. Chem.* **35**, 1043–1052. 10.1016/s0223-5234(00)01189-2 11248403

[bb7] Kappe, C. O., Fabian, W. M. F. & Semones, M. A. (1997). *Tetrahedron*, **53**, 2803–2816.

[bb8] Rovnyak, G. C., Atwal, K. S., Hedberg, A., Kimball, S. D., Morebend, S., Gougeutar, J. Z., O’Reilly, B. C. & Malley, M. F. (1992). *J. Med. Chem.* **35**, 3254–3263. 10.1021/jm00095a023 1387168

[bb9] Rovnyak, G. C., Kimball, S. D., Beyer, B., Cucinotta, G., Dimarco, J., Gougoutas, D. J. & Moreland, S. (1995). *J. Med. Chem.* **38**, 119–129. 10.1021/jm00001a017 7837222

[bb10] Sheldrick, G. M. (2008). *Acta Cryst.* A**64**, 112–122. 10.1107/S0108767307043930 18156677

[bb11] Shivarama Holla, B., Sooryanarayana Rao, B., Sarojini, B. K. & Akberali, P. M. (2004). *Eur. J. Med. Chem.* **39**, 777–783. 10.1016/j.ejmech.2006.02.001 16616396

[bb12] Spek, A. L. (2003). *J. Appl. Cryst.* **36**, 7–13.

